# “They’re all struggling as well”: social and economic barriers and facilitators to self-managing chronic illness among marginalized people who use drugs

**DOI:** 10.1080/17482631.2022.2082111

**Published:** 2022-06-02

**Authors:** Lisa M. Boucher, Esther S. Shoemaker, Clare E. Liddy, Lynne Leonard, Paul A. MacPherson, Justin Presseau, Alana Martin, Dave Pineau, Christine Lalonde, Nic Diliso, Terry Lafleche, Michael Fitzgerald, Claire E. Kendall

**Affiliations:** a School of Epidemiology and Public Health, Faculty of Medicine, University of Ottawa, Ottawa, ON, Canada; b C.T. Lamont Primary Health Care Research Centre, Bruyère Research Institute, Ottawa, ON, Canada; c Department of Family Medicine, Faculty of Medicine, University of Ottawa, Ottawa, ON, Canada; d Clinical Epidemiology Program, Ottawa Hospital Research Institute, Ottawa, ON, Canada; e Department of Medicine, Faculty of Medicine, University of Ottawa, Ottawa, ON, Canada; f Somerset West Community Health Centre, Ottawa, ON, Canada; g Centretown Community Health Centre, Ottawa, ON, Canada; h The CDSM among PWUD Study’s Community Advisory Committee, Bruyère Research Institute, Ottawa, ON, Canada; i Sandy Hill Community Health Centre, Ottawa, ON, Canada

**Keywords:** Self-care, chronic disease management, drug use, socioeconomic marginalization, relational autonomy, community-based research

## Abstract

**Purpose:**

Self-management is recommended for addressing chronic conditions, and self-management programmes improve health behaviours and outcomes. However, social and economic factors have been neglected in self-management research, despite their relevance for marginalized groups. Thus, we aimed to explore barriers and facilitators that influence self-management among socioeconomically marginalized people who use drugs (PWUD).

**Methods:**

Using community-based participatory methods, we developed a qualitative interview guide and conducted peer-led recruitment. Participants were admitted into the study after self-identifying as using non-prescribed drugs, having a chronic health issue, and experiencing socioeconomic marginalization. Data were analysed using reflexive thematic analysis, taking a relational autonomy lens.

**Results:**

Participants highlighted substantial barriers to managing their health issues, mostly stemming from their social and economic environments, such as unstable housing, low income, lack of supportive social networks, and negative healthcare experiences. Participants also described how their ability to self-manage their chronic conditions benefited from specific aspects of social interactions, including close relationships, community connectedness, and engaging in peer support.

**Conclusions:**

Our findings suggest that structural interventions are needed to support self-management among marginalized PWUD, especially stable housing. Self-management supports for PWUD would benefit from including a range of low-barrier community-based options, peer work opportunities, and advocacy for needs.

## 1. Introduction

People with low incomes tend to be negatively impacted by additional social determinants of health, which drive health inequalities including heightened morbidity and mortality (Public Health Agency of Canada, 2018). For many people who use drugs (PWUD) illicitly, who often experience socioeconomic marginalization, these poor outcomes are exacerbated by the complexity of their health and social issues (Kreek, 2011; Richardson et al., 2015). In addition to their drug use, common long-term conditions include chronic pain, mental health conditions, and infectious diseases (Dassieu et al., 2019; Kendall et al., 2017). Many PWUD also identify with multiple marginalized groups, thus often facing intersectional stigma, for example, due to racism, heteronormativity, poverty, criminalization, drug use, and stigmatized health issues (Boucher et al., 2017; Logie et al., 2011).

Given evidence that self-management programmes improve health outcomes across many chronic conditions, self-management services are growing within health and social care domains (Allegrante et al., 2019). Self-management can be defined as “the tasks that an individual must undertake to live well with one or more chronic conditions. These tasks include gaining confidence to deal with the medical management, role management and emotional management of their conditions” (Adams et al., 2004). As most chronic illness self-management occurs in people’s day-to-day lives, outside of the purview of healthcare, tailoring self-management supports to suit their everyday contexts is an important consideration. Yet, it is unclear whether the current body of self-management support evidence applies equally to marginalized groups, and specifically PWUD. A number of authors have identified gaps in the literature on chronic disease self-management initiatives, in particular a lack of attention to social and economic conditions, which may contribute to lower availability, accessibility, or acceptability of self-management supports among people with socially complex needs (Goodridge et al., 2019; Kennedy et al., 2007; Newbould et al., 2006).

While changing individual health behaviours has been the prominent focus of many self-management supports, there is growing emphasis on the need to target the social elements of self-management (e.g., social networks, social capital), especially with attention to socioeconomically marginalized groups (Goodridge et al., 2019; Morris et al., 2016; Rogers et al., 2011; Tausig, 2013; Vassilev et al., 2013, 2011). For instance, people living with HIV have highlighted social support and stigma as important aspects of their self-management (McDonald et al., 2016), yet participating in social activities and roles has been a neglected outcome in self-management research (Packer et al., 2018). To facilitate addressing social needs as well as stigma, peer-led self-management interventions may be most suited to marginalized communities.

Many of these critiques share a concern that current chronic disease self-management initiatives may inadvertently contribute to adverse outcomes for certain populations (Kendall et al., 2011). That is, unless they increase attention to removing barriers for the most marginalized groups experiencing chronic conditions, these initiatives may further disadvantage people who need the most support. Accordingly, the International Framework for Chronic Condition Self-Management Support has identified health equity as one of four priorities for advancing the field, noting that the reach, range, and access of self-management support needs to be expanded (Mills et al., 2016). This framework and other researchers highlight the need to explicitly target self-management programmes to disadvantaged groups to avoid exacerbating health inequities (Packer et al., 2012; Mills et al., 2016).

To investigate this largely unexplored topic and determine how interventions may be best tailored for this population, we required an in-depth understanding of current self-management experiences among PWUD. We sought to assess the social and economic factors that promoted or interfered with the capacity for self-management among PWUD by asking the question: *What are the barriers and facilitators to self-managing chronic health issues including drug use among marginalized PWUD?*

## 2. Theory

One suggestion for addressing some of these critiques has been to incorporate the concept of *relational autonomy* into chronic illness self-management (Ould Brahim, 2019), as has been suggested for other popular approaches to improving healthcare, such as patient-centred care (Ells et al., 2011), and even specifically to improve mental health services for people with substance use disorders (Lago et al., 2020). A relational conception of autonomy contends that “an analysis of the characteristics and capacities of the self cannot be adequately undertaken without attention to the rich and complex social and historical contexts in which agents are embedded” (Mackenzie & Stoljar, 2000), with particular importance placed on analysing how oppressive social contexts undermine autonomy. This is opposed to the traditional, individual or personal view of autonomy, which “ … neglects social and material circumstances and the power relations that impact choice, agency, and selfhood” (Ould Brahim, 2019). Thus, a relational autonomy lens can contribute to addressing challenges that arise from the typical dichotomy between agency and structure in the social sciences, which also tends to characterize discourse on marginalized groups in both policy and academic arenas (Clapham, 2007). That is, the single concept of relational autonomy highlights the intersection between people’s environmental context and their individual behaviour; thus, it can be used to delineate these complex interactions and unify the sets of factors related to structure versus agency into a fuller understanding of phenomena.

Within the self-management domain, Ould Brahim (2019) highlights the critical importance of autonomy along with the limitations of current interventions, including the way that common self-management programmes (similar to other initiatives focused on health behaviour change) tend to neglect the pertinent influences of structural and social factors. Thus, this author proposes that a relational view of autonomy can address many of the existing issues within such initiatives through focusing on environmental factors, advocating for systems-level change, and engaging with a “patient nexus” (including consideration of how behaviours are constrained or supported by social relations, especially class). In this study focused on socioeconomically marginalized PWUD, we drew on relational autonomy in conceptualizing our understanding of self-management, as this approach was particularly important given that limited attention to structural factors is likely to disproportionately harm marginalized groups as well as misrepresent their experiences. This approach allowed us to avoid over-emphasizing the influence of internal individual characteristics on behaviour. Rather, we considered the social and economic circumstances and power relations that affect individuals’ self-management, and considered social-ecological models and their emphasis on “interdependence between people, their behaviour, and their environment” (Ould Brahim, 2019) in developing our interview guide, codes, and themes.

## 3. Materials and methods

The Bruyère Research Institute and the University of Ottawa Research Ethics Boards approved this study (numbers M16-19-027 and H-10-19-5175, respectively), which was conducted in Ottawa, Ontario, Canada. In this North American setting, there has been an opioid overdose crisis affecting PWUD for many years. Drug use is criminalized and drug policies have remained mostly punitive, despite increasing recommendations from experts (e.g., researchers, service providers, decision-makers) for decriminalization and safe supply to stem the worsening toxicity of the street drug supply and resulting death toll (Ivsins et al., 2020). Yet, compared to some other countries (especially our closest neighbour, the USA), Canada has well-established public health support for various types of harm reduction initiatives (e.g., needle and syringe programmes, supervised consumption services, naloxone distribution). Such progress has typically been attributed to early leadership among drug user activists in Vancouver, British Columbia (e.g., Insite has been open since 2003 and was the first supervised injection facility in North America). Further, Canada made global strides in progressive drug policy by becoming the second country to legalize cannabis recreationally nationwide in 2018. In addition, in our setting there is currently an affordable housing crisis, which was declared locally in the city of Ottawa in January 2020 (Osman, 2020). With respect to welfare policy in our setting, social assistance or disability payments are available from the province of Ontario to eligible individuals, however the amounts provided remain below the poverty line and restrict formal labour force participation. The disability benefits also provide people with prescription drug coverage, and Canada offers universal public health insurance for core services (e.g., emergency or hospital admission, physician visits). Despite all these benefits, among the wider public, non-prescribed drug use remains highly stigmatized, which affects many aspects of life for PWUD, including limiting the accessibility of healthcare (e.g., pain management) and social services (e.g., housing supports).

In this study, we established a community-academic partnership through community-based participatory research (CBPR) (Israel et al., 2010). CBPR emphasizes the meaningful participation of people with lived experience, as well as co-learning and acting for social change (Flicker, 2005), with expert consensus highlighting the importance of engaging community members in self-management initiatives (Mills et al., 2016). Despite the notion that patients are key experts on chronic disease self-management (Lorig, 2002), those who are marginalized are often left out of decision-making that affects them. Thus, we employed a transformative framework to help amplify the voices of this marginalized group and support addressing social inequities (Creswell & Poth, 2018), which is compatible with the focus of CBPR on acknowledging the value of experiential knowledge (Flicker, 2005).

The lead author had experience working with the study population in previous participatory research studies and ensured community members were engaged throughout the study process. There were no conflicts of interest within these working relationships (e.g., the lead author was not a healthcare or social service provider and thus did not have perceived or actual control over participants’ access to care). The Community Research Coordinator had lived experience of drug use and socioeconomic marginalization, as did four other community members selected for the Community Advisory Committee (CAC). They were selected for having different levels of past research experience, representing diverse social positions and experiences in common with the study population, and being engaged with community organizations in various capacities. CAC members provided guidance on methods, developed data collection tools, conducted recruitment, supported analysis and interpretation, and strengthened knowledge translation. Members signed confidentiality forms and were compensated for their work.

We developed a semi-structured interview guide through extensive CAC discussions, with focused attention on identifying the most appropriate language for the community. For example, we decided to ask participants plainly what factors influenced how they “manage” their chronic conditions, and to use the term “self-care” as it was more familiar to the community than “self-management”. Questions focused on barriers and facilitators to self-managing chronic conditions, including relevant supports, such as “What makes it harder for you to manage your long-term health issues, including/or your drug use? What makes it easier?” and “What supports or services do you receive to help you manage your chronic health issues?” We also developed a list of prompts to ensure we could explore the relevance of certain factors (e.g., stigma, social networks).

CAC discussions resulted in narrowing the eligibility criteria to focus on participants who self-identified as having: 1) past year and long-term use of non-prescribed drugs (i.e., use of drugs obtained illicitly or prescription drugs not as directed, but not including only cannabis); 2) at least one other chronic health issue; and 3) current financial difficulties. We used the maximum variation purposeful sampling strategy to identify important differences in perspectives (Creswell & Poth, 2018), with the CAC considering relevant factors to include: sex, gender, sexual orientation, age, ethnicity, typical health issues and drug use patterns in the community, typical marginalizing experiences (e.g., housing, sex work, incarceration), and degree of engagement in services.

Our recruitment process was based on a successful street-based, peer-led approach employed in prior qualitative research among this community (Boucher et al., 2017). The CAC identified “hot spot” locations and three different community researchers led recruitment. This process capitalized on community researchers’ access to marginalized PWUD while also facilitating introduction and rapport for the lead author with participants. To reduce non-attendance, recruited individuals were mostly scheduled for an interview within a few hours. Given that interested participants had to identify as engaging in stigmatized activities and some of our questions were sensitive, we were careful to assure potential participants that their responses would remain confidential, and we purposely refrained from asking specific details about certain topics (e.g., criminal behaviours or traumatic experiences).

We conducted in-person, one-on-one qualitative interviews and compensated participants CAD 30.00 for their time. Data collection and analysis were iterative, allowing ongoing refinement of the interview guide and recruitment strategy. Four community-based organizations were identified as preferred sites to conduct interviews because many marginalized PWUD frequent them in our setting and find them to be welcoming spaces. Further, these locations were chosen because they contain many supports specifically targeted to this population (including low-barrier, drop-in supports and counselling), thus in the event of a crisis during data collection there would be support available to participants. We also created and provided a list of community resources to interested participants. The lead author conducted all interviews, obtaining prior written informed consent and administering a brief sociodemographic questionnaire subsequently.

Interviews were audio recorded and transcribed verbatim, then transcripts were coded by hand and within NVivo software (QSR International Pty Ltd, 2013). We used reflexive thematic analysis (V Braun & Clarke, 2006; Braun & Clarke, 2019a, 2020), a qualitative research approach in which both manifest and latent content are considered during data analysis, with patterns and threads identified across the data. Two academic and one community researcher collaborated to conduct coding, with the community researcher also providing cultural interpretation. Codes and themes were then brought to discuss with other academic and community members of our team, with adjustments made to reflect the insights gained. We generated all codes and themes through an inductive approach, rather than identifying any in advance. A focus on data saturation was not conducive to our analysis and instead we applied the concept of information power, which is supported by our focused study aim, theory-guided investigation, and data containing strong quality dialogue (Braun & Clarke, 2019b; Malterud et al., 2016).

We considered trustworthiness criteria to improve rigour and quality in our methods (Guba, 1981). To increase validity, we adopted line-by-line coding and constant comparison (Creswell & Poth, 2018). In addition, we used the following strategies: acknowledged our personal biases and assumptions (e.g., examined social positions and power); collected rich contextual details to facilitate assessing how applicable findings are to other contexts; and used thick quotes to allow readers to make their own judgements (Noble & Smith, 2015; Shenton, 2004). Pseudonyms are used to maintain participant confidentiality.

## 4. Results

Interviews were an average length of 1 hour and 13 minutes. Our sample included 15 participants with an average age of 45 years (range = 27 to 70), 47% male, 67% heterosexual, and 60% white. In the past year, 93% received either disability or social assistance payments, 53% worked part-time or casual jobs, 67% received some money from family or friends, and 73% obtained street-based income (i.e., sex work, drug dealing, panhandling, selling handmade items, other activities). Further details about the sample characteristics and self-management strategies are reported separately (Boucher et al., 2022), including that most participants considered their drug use to be a chronic health issue. All had extensive experience using stimulants (e.g., crack/cocaine, crystal meth), opioids (e.g., heroin, fentanyl), or both. Participants had many other chronic conditions, including chronic pain, mental health issues, infectious diseases, and other physical health issues. Acute and recurrent health issues were also experienced frequently.

We identified three themes expressing the nature of key barriers and facilitators that influenced how marginalized PWUD were able to self-manage their chronic health issues, including with respect to meeting basic needs, navigating social networks, and accessing healthcare services.

### 4.1 Difficulty meeting basic needs interfered with managing chronic health issues

Participants described the ways in which limited access to the material means (e.g., shelter, food, transportation) necessary to meet basic needs interfered with their ability to develop or maintain self-management behaviours and routines. When participants experienced unstable housing or inadequate income, they had to spend much time engaged in survival activities, which were sometimes all-consuming and caused further harm to their mental and physical health. Thus, participants were often unable to address less urgent needs, such as their chronic health issues.

#### 4.1.1 Unstable housing meant lacking a foundation for self-management

The most prominent barrier to self-managing health issues mentioned by participants was an unstable housing environment. They experienced stress due to other people in or near their living space (e.g., people staying/living in their room/building; support staff or building managers) or due to the conditions of the housing or shelter (including lack of space, accessibility, or safety), which worsened their health issues, especially mental health and drug use. For instance, Jeremy highlighted how being homeless contributed to increased stimulant drug use and the inability to maintain a healthy sleep pattern: *“We’re deprived of sleep always. And when you are able to sleep, it’s hard to remain in a very restful state because you’re so used to being woken up or have someone out there who’s preying on you for something.”*

Jeremy further explained how homelessness led to recurring acute health issues and interfered with the ability to manage them well enough to avoid progression to chronic issues:

*… everyone I know has issues with their feet … you don’t often have an opportunity to have the proper foot gear … so many of us end up travelling long distances with you know, wet socks or very cold feet … And staying hygienic, right, is difficult sometimes. So, if you have a tiny scrape or wound, it can get far more, you know, complicated than it normally would.*

Consistency or regularity in daily activities was identified as a key mechanism through which stable housing worked to support self-managing chronic health issues:

*… having a home, especially, you know, you tend to fall into routines and patterns and go to bed at a certain time or take your medication at a certain time. So, things were much more regimented, and now it’s very random. Yeah, so that’s really the main thing is that there’s no consistency to how I eat or sleep or maintain my health in any way. … Like, in terms of my HIV especially, I’d like to take my medication regularly, see my doctor regularly, have blood work done regularly. (Jeremy)*

Similarly, Cynthia described how finally securing stable housing was the pivotal event that allowed her to start self-managing her chronic health issues: “*But that was one of the first things that allowed me to begin any kind of self-care. … And then I started to be doing things like, you know, I had real problems because of the circulation. I had to go to the chiropody for about a year where they were caring for my feet … ”*

Participants also explained how the common experience of losing their housing interfered with progress they were making in managing their health issues: “ … *just when I was starting to get up there … and I went down again, you know, just when I was starting to feel really good and strong and I was having a somewhat regular, almost normal routine.” (Rebecca)*

Many participants expressed a strong desire to obtain better housing, with several indicating that it would substantially improve their ability to manage their health issues including drug use, as Rebecca said: “*Oh, I wish I had my own place. I really believe if I had my own place I would be able to quit everything.”* Thus, overall participants spent a lot of effort strategizing how to maintain or improve their housing, limiting how much they could focus on managing chronic health issues.

#### 4.1.2 Insufficient income restricted self-management options

Having inadequate income, and consequently the need to figure out ways to obtain enough income to meet their basic needs, was common among participants and a barrier to managing their health issues. Some participants attributed certain health issues to a lack of access to health services due to their low income. For example, health issues which might have been resolved quickly through obtaining healthcare sometimes developed into long-term concerns, as Jacob described with respect to dental issues: *“I have really bad teeth problems because I’m too broke, I’ve been broke for awhile. … they’re literally like chipped and my root canal fell out … ”* Likewise, low income often resulted in a lack of access to transportation, which negatively impacted participants’ health and self-management practices. Because many relied on community services for meals, warmth, hygiene, or other basic needs, they often had to traverse large areas of the city by foot, contributing to ongoing feet problems.

Participants highlighted how finding ways to obtain enough income to meet their needs (including to buy drugs) was critical for managing their health issues, yet the continual searching that this involved consumed much of their time and interfered with other self-management activities. This was especially true among participants who were using drugs more heavily. Many inventive methods were employed, with illegal activities typically a last resort. The need to engage in undesirable activities caused participants stress, which in turn became a barrier to their self-management. For instance, participants who engaged in survival sex work described it as troubling and linked to their drug use: “*Well, it was necessary to do the drugs in order to, you know, do the sex work, in my case at least. And I found that without it, I wasn’t really able to, you know, it’s not something I could bring myself to do unless I was high. … but it’s sort of a vicious cycle.” (Jeremy)*

On the other hand, receiving income support (e.g., disability or social assistance, money or items from family or friends) or financial management supports (e.g., direct rent payments) was a facilitator for managing health issues. A few participants also expressed how fortunate they felt in being able to access additional benefits, which for Cynthia made the difference in being able to afford items to address her chronic health issues: “ *… I do have compression stockings. … They paid for them because I’m on ODSP but they’re like 100 bucks a pair. I would have never been able to afford it.”* Similarly, Melissa described how her supports were important for managing unstable drug use:

*I have a bank account at [a community organization] for the money management. I still need help on different angles and that because it’s like it doesn’t matter if I don’t have the money … it seems like the drugs come my way. … Now I have to report it back to my worker because now they’re doing a budget with me.*

### 4.2 Navigating social networks presented challenges and opportunities for managing chronic health issues

Participants’ social networks had a critical influence on how they managed their health issues, both through their personal social interactions (e.g., with partners, family, friends, peers, and pets) and through engagement with community services. Overall, participants’ social environments presented challenges which they had to navigate cautiously to manage their own health issues, especially with respect to mental health and drug use, sometimes leading to self-isolation and lack of connection. Community supports and peer work opportunities helped fill the gaps in PWUD’s social networks.

#### 4.2.1 Need to be careful trusting others

Many participants described histories of negative interpersonal experiences, including abuse or betrayal and the loss of loved ones. Such experiences led them to have difficulty trusting others and interfered with forming or keeping close relationships, with most having at least some ongoing conflict with family or friends. Some expressed having a lack of relationships overall: *“I have nobody else in my life but myself.” (Melissa)* This lack of social ties harmed participants’ health, especially their mental health and drug use. The influence was especially pronounced with respect to being estranged from their children: “*And I have a daughter by the way. … So I haven’t seen her in a while, so that’s also a different health problem, I guess, mental problem*. … *It affects everything. … Yeah, like depression hits hard when it hits, especially at times like Christmas.” (Michelle)*

Furthermore, participants described interacting with many other marginalized PWUD, which made them wary about trusting or relying on others:

*… it’s so hard to trust people in the scene so, you never really know who’s a real friend and who isn’t. … It can be difficult to manage those types of relationships and figure out who’s, you know, with you because you have access to drugs or because you have something that they want or need. (Jeremy)*

Participants found that these social environments made it hard to avoid using drugs, especially around “cheque day” (i.e., when people receive monthly government income assistance). Some also faced pressure to sell drugs or commit other illegal acts. In housing environments where there were a lot of PWUD (e.g., shelters, rooming houses, some social housing), it was especially difficult to avoid these influences. On the whole, these environmental stressors led to unstable drug use and decreased self-care for health issues. For instance, Anthony noted how being entrenched in the social environment of drug using and selling interfered with managing his health: “*What doesn’t make it easier is I have easy access to drugs. … Because it’s been a part of my lifestyle for awhile so any drug I want I can easily get my hands on and so that does not help my health issues.”*

To manage these social challenges, some participants chose to have few close relationships. For instance, some felt the need to isolate when they were using drugs or to hide their drug use from specific others: *“But good friends I don’t access them enough, you know like I … I don’t want them to know what’s really going on.” (William)* While this lack of connection was at times upsetting, at other times it helped participants focus on managing their own health issues. For instance, especially among women participants, intimate relationships could present challenges to managing health. As Michelle noted, her current relationship exacerbated her mental health issues and drug use: *“So like that’s a big stressor and that’s one of the main reasons I use a lot more than usual.”* Some women reported not being interested in romantic relationships because they were managing better on their own. Melissa, who had experienced domestic abuse and serious mental health issues, expressed a strong desire to abstain from such intimacy: “*I’m finding that I’m pretty stable now because I don’t have no boyfriend or no girlfriend … No. I don’t want no relationships.”*

#### 4.2.2 Obtaining social supports to meet emotional and practical needs

Most participants reported having at least some social support that helped with managing their long-term mental and physical health issues. Having either a few close relationships or a larger social network contributed to improved self-management, as did being connected to one’s community in general. While the main types of social supports participants described were emotional ones, they also received practical supports from others, including financial contributions, access to resources or items, and information or assistance with completing tasks, although such supports were comparatively rare.

Among participants who had them, positive romantic partners were another source of support in managing their health issues, especially among men in our sample. Several described having deeply supportive significant others:

*Anything I ask her to do she does. She goes and gets the bandages for my legs. … I don’t know too many of the resources. Like my girl takes care of all that stuff and I got a pretty bad memory too so. (Brian)*

Likewise, as Jacob explained, feeling that someone truly cared helped him use drugs in less detrimental ways, while also motivating him to improve basic self-management behaviours such as nutrition:

*… since [my girlfriend]’s come into my life I haven’t been fucked up with opioids to the point where I’m like, you know, bumping into shit, and with benzos I haven’t been blacked out … now that I have someone that like genuinely cares about me and isn’t just trying to get something out of me, you know, it feels really good … Me and [my girlfriend] both have only started to eat since we’ve been together because we’re like – reasons to care for yourself is for the other person, right? So like I’ve been eating a lot more since – I used to eat basically like the cheapest things …*

Similarly, some participants described an immense positive influence on their mental health and self-care activities from close friendships. As Rebecca described, her friend provided both emotional and practical support:

*But I finally found somebody who I can trust, and she’s really helped me a lot, and I feel mentally a lot stronger now … And she’s really creative, and she’s very warm and everything … She really encourages me, yeah, to do all the things I want to do, and she really gets me started …*

In contrast, other participants’ most critical social supports stemmed from being well-engaged with certain services in the community, including with community workers (e.g., case managers, mental health workers) as well as entire organizations that provided a plethora of support options. For instance, participants highlighted how specific service providers had a huge impact on their progress towards improved self-care:

*Like she – people are so empathetic and understanding and supportive and it’s – those things are essential because even if you want to make change in your life … you can’t see a way there until somebody starts saying here, come with me, I’ll put you on that path, you know, I’ll nudge you over here, I’ll lift you up here or I’ll drive you here or, you know, get you involved in this … (Cynthia)*

When asked about whether they had community support or felt like they were part of a community, participants often interpreted this as referring to community organizations that they engaged with and which helped them in a multitude of ways: *“ … it’s like my second home, I’m here so often.” (Scott)* Low-barrier, one-stop-shop style supports were particularly helpful. Several participants also highlighted how their preferred community organizations encouraged their input on how to best offer supports. Moreover, feeling connected to community motivated participants to deal with their health issues in improved ways, as in this example in which engaging in community activities directly helped Eric improve his drug use:

*It was a pot luck and drumming and Aboriginal gathering. I chose to go to that because I didn’t want to use that day, because it was cheque day and I had a pocketful of money and I’m like, well I want to do something different today. And I did. I really enjoyed it. It was quite fun. It didn’t make me think about using or anything, a positive group of people and we had a great time.*

Similarly, when asked what helped her to cope with her health issues, Michelle described how panhandling made her feel more connected to the broader community: “*I go panning. I’m a people person, I like people. … and it helps me talk to somebody who I don’t know and just talk to say hi, and they stop and talk to me and it makes me feel like you’re more wanted, I guess in a way, and you’re not being judged by certain people.”*

Participants also expressed the importance of receiving peer support through community organizations. They described preferences for different types, with some preferring one-on-one and others group supports, as well as specific or innovative ideas for how it should be offered: “*I wish there was more—like a mentorship program … Kind of like what AA people, in AA have, you know? … But like something more for opioid users, you know some kind of people who have been through that and have overcome it.” (Rebecca)*

Nevertheless, some participants were connected to few or no community supports, despite recognizing the value of such engagement and desiring to pursue more connectedness.

#### 4.2.3 Supporting peers and others

In addition to the benefits of receiving social support from others including their peers, participants highlighted how much they benefited from providing support to others. This included working in formal peer worker roles, as well as informal support they often provided for friends, family, pets, and acquaintances in the community. Engaging in such helping activities enhanced participants’ social networks and sense of community connectedness, reducing isolation and increasing their desire or capacity to engage in self-care and continue caring for others. While participants engaged in many different forms of helping, most desired more paid peer work opportunities, given that their precarious financial situations made it challenging to engage in unpaid activities.

On the whole, participants emphasized the powerful nature of peer support among PWUD, including how a positive feedback loop occurred through helping one’s peers, as Jacob described with respect to his volunteer peer support role:

*So it’s like a user base kind of, we’re all helping the community, trying to like help each other, right? … that’s been the best support system just because it’s about drugs and addiction and I can talk to them and they understand. … the best form of recovery and the thing that works, and has been proven to work the best, is addicts helping other addicts.*

Michelle expressed that being involved in creating and providing formal peer supports made her feel accomplished, which motivated her to take better care of herself: *“So women had a place to drop in between that time out of the cold in the winter especially. So yeah, we did that for, I did that for a year and a half. And I was one of the ones that initiated it. So it felt good. So stuff like that it makes me want to, you know, do better.”*

However, while many participants were generous in trying to help other people who were also struggling, the added stress from doing so often risked their own self-management: *“I used to take the odd person off the streets, take them home with me … With their mental health stable and my mental health stable and that, you just can’t deal with it. … Because you’re only going to bring yourself down. So now I don’t bring nobody home … ” (Melissa)* While sometimes PWUD were taken advantage of when they tried to help others, on other occasions their mental health was harmed when their efforts to help others did not succeed: *“So I promised myself no more of that, because they just stressed me out, so yeah. … I get stressed out because I can’t help them. … I don’t want to feel any worse than I do about not being able to help my friends.” (Mark)* Thus, many participants came to the conclusion that they needed to prioritize their own self-care before they could help others: “*I used to always be like how I showed somebody I cared for them was to do everything for them and then I took a backseat myself and anything to do with my own life took a backseat. … but I have no problem prioritizing myself now. … So I feel good about that … ” (Cynthia)*

Similarly, when asked whether his peer group was supportive, Jeremy provided a few reasons why it was challenging to maintain mutual support amongst marginalized PWUD:

*As much as they can be, but they’re all struggling as well, right? They’re all dealing with their own emotional and mental fragility, you know, so it’s hard to be a real support to each other. … you’re not always able to be in contact with them because we don’t necessarily have phones. … getting to a computer to use email is difficult. … So, communication is really tricky when you’re living this way. … And knowing where someone might be at any given time is almost impossible.*

Furthermore, a participant who was an employed peer worker emphasized why it is important to increase the extent of paid peer positions, rather than expecting marginalized PWUD to rely solely on support from their social networks, which largely consist of other people who are struggling:

*Because I see many people, and especially those people that are in crisis that I’m helping … completely alone in life, like most people don’t have anybody but other badly bent people. … because most people even if they have friends that are in the same boat as them they’re not in a position to really do much to help. (Cynthia)*

### 4.3 Negative experiences accessing healthcare services limited chronic illness self-management support options

Participants described how they needed to access many different health and social care services to manage their chronic health issues. While community supports were typically helpful, more traditional healthcare services presented problems for PWUD. Participants often highlighted unmet healthcare needs due to inadequate access or negative experiences. Access issues related mainly to socioeconomic and systemic healthcare conditions. Lack of access to primary care and specialist providers and to certain medications was noted, as well as lack of or misinformation, difficulty getting to services (e.g., proximity, transportation, weather), and a lack of low-barrier services to address their multiple healthcare needs. Participants with complex health issues reported finding it hard to obtain a regular doctor:

*Every time I call that Telehealth thing they call me back with an appointment with a doctor and then they look at the list of the medication I’m on and they turn around and call back a day or two before saying that they can’t help me and they’ll see if they can find me another doctor. It’s been going on for two years*. (Brian)

While many of participants’ negative interactions resulted from discrimination, some may have been related to other systemic issues, such as inadequate training or resources. For example, participants who injected drugs experienced challenges in having blood taken because it took providers many tries to find a vein. Many participants also described unpleasant experiences with psychiatrists or psychiatric medications. While several had positive experiences with counselling, others were not comfortable with it and some even found it interfered with managing their mental health issues, as in Amy’s example:

*… it’s dug up more pain than I can manage … Social workers, psychiatrist, counsellor, psychologists … Just picking away and thinking they’re doing more good, but it’s actually making the problems come out a lot more, a lot faster, and a lot harder. … and when you’re talking about this stuff, they’re only available when they’re available, but when they open wounds, they can’t just stitch them up whenever they can. … You leave the wounds stuck open, then I’m open and I’m vulnerable, and I have no idea what to do with myself. Do I go get more help or do I feel like killing myself or what?*

Overall, the stigmatization of PWUD was the greatest barrier participants reported in accessing healthcare to support managing their health issues. Given lack of primary care access, participants often had to seek care in hospitals, which resulted in highly negative experiences that led to future healthcare avoidance: *“As soon as they see my history they are so judgemental and so rude and just cold and mean. That’s the worst place. Even if I’m really sick it takes a lot [to go there].” (Rebecca)*

However, while many participants encountered difficulties with advocating for themselves, a few highlighted the importance of advocacy as a facilitator for self-management in interactions with healthcare providers. Cynthia described her need to self-advocate to get support for managing a chronic health issue during an encounter in which she felt she was stigmatized against based on her drug use:

*I didn’t feel I had a lot of support from my [specialist]. Like anything I learned about my condition I did not learn from her and every time I asked for help or asked to be seen or whatever, like I mean she really literally was not doing anything. … And when I went back to her I said, you know, the first day I met you and described my history of addiction I saw a look pass over your face that I’ve seen many times, that has to do with those biases and stigmatism and I said I don’t know if you’re aware of it. But anyways meanwhile I’m like needing somebody, I don’t know if we’re going to go further together on this or if you’re going to refer me to somebody but I am now putting you on notice …*

## 5. Discussion

In this study, we attended to several important gaps in the chronic disease self-management literature, facilitated by our relational autonomy approach to self-management (Ould Brahim, 2019). In light of the critiques of self-management initiatives for failure to consider the social embeddedness, access to economic resources, and power dynamics that shape people’s lived realities, we investigated barriers and facilitators that influence the ability of marginalized PWUD to self-manage their chronic health issues. Our findings demonstrate how challenging social and economic environments constrain self-management, as participants experienced many more barriers than facilitators, which all correspond to well-known social determinants of health.

The most persistent impediment to managing chronic illness occurred when participants did not have their most basic needs met. Stable housing was foundational to developing and maintaining self-management practices in PWUD’s daily lives, as many negative housing and shelter-related experiences interfered with managing their health issues, including by worsening their drug use. Most expressed that more stable housing would allow them to improve how they manage their health issues. They spent substantial effort looking for better housing or maintaining current housing, which also interfered with prioritizing health issues. The prominence of housing concerns in this study was not surprising and highlights the importance of providing permanent supportive housing to facilitate stability as a basis for self-management, adding to evidence for Housing First among PWUD (Palepu et al., 2013). Further, participants discussed how they could not focus on managing long-term health issues due to inadequate income and time spent finding various ways to obtain enough income to meet their needs. Together, both unstable housing and low income prevented PWUD from developing consistency in their daily routine, which was critical for their chronic disease self-management. In addition, these socioeconomic challenges contributed to more acute health issues which necessarily took precedence over chronic health issues, and sometimes even became chronic when unaddressed. This relates to participants’ frequent use of emergency healthcare services versus primary care services, corroborating previous findings with this population (Kendall et al., 2020; Kendall et al., 2017).

Another critical theme was social interactions, which either hindered or supported participants’ chronic disease self-management. This is unsurprising, given the influence social networks have on self-management (Vassilev et al., 2011). Marginalized PWUD must navigate challenging social environments that include many other PWUD and people with low socioeconomic status. Participants often expressed difficulty trusting others and chose to isolate themselves to avoid relying on others. Such choices typically related to their past negative social interactions, including traumatic experiences. On the other hand, some participants described receiving immense emotional support from romantic partners or friends with whom they had formed close relationships. Participants were extremely grateful when they received this high level of support, expressing how it was rare in the context of their lives. In some cases, professional community workers provided emotional support that filled gaps for participants who had very limited or no close personal relationships, and participants were similarly grateful when this occurred. Some participants received practical supports as well from family and friends or workers, but this was less prominent. Finally, participants also described reciprocal benefits from peer support and strong desires to help their peers and others, often attempting to do so informally until it became too difficult to manage their own issues. The scarcity of resources and complex needs of other people in the lives of marginalized PWUD demonstrates the critical detriment of a low-resourced social network (Tausig, 2013). Thus, it is not surprising that many participants also expressed that the potential to work in paid peer support positions would resolve many issues they encountered, allowing them to help both others and themselves, as research on low-barrier employment opportunities has found (Penn et al., 2016). Those in formal peer support roles further expressed the benefits for their own health issues, also corroborating previous research (Watson, 2017).

These findings add to the literature on the importance of social networks in self-management, and our study meets the call of these authors to explore the generic ideas among specific populations and contexts. While people with chronic conditions tend to get most of their social support from close family (e.g., adult children and spouses), many participants in our study did not have these types of relationships (Vassilev et al., 2013). Our findings appear to suggest a critical lack of strong ties and a predominance of weak ties (although some participants lacked even weak ties), and weak ties may not be as helpful for chronic illness management as they are in other areas of people’s lives (Morris et al., 2016). Further, our findings around PWUD’s positive emphasis on peer support and low-barrier services, along with their negative healthcare experiences, highlights potential benefits of shifting the emphasis to self-care supports outside of formal healthcare, aligning with previous findings (Rogers et al., 2011). However, as evidenced by our participants’ experiences, social networks and communities must be supported and resourced to do this work, specifically among PWUD and other marginalized groups where many people are similarly suffering.

Finally, while most participants highlighted benefiting from low-barrier community services, we found some evidence that PWUD’s challenges in accessing healthcare services interfered with their chronic disease self-management. In addition to typical barriers to healthcare access, such as transportation, participants faced extensive stigma based on their drug use, which is well-documented in the literature (Biancarelli et al., 2019; Paquette et al., 2018). For instance, participants felt discriminated against by doctors when they did not receive adequate pain medication. This is in line with recent research among PWUD with chronic pain (Dassieu et al., 2019), highlighting how common practices of prioritizing substance use in clinical care for PWUD can lead to their other health issues being interpreted through a substance use lens, leading to reduced access to other care. Further, PWUD’s negative past experiences with healthcare services made them less likely to trust providers and more likely to avoid them in the future, as other studies have found (Biancarelli et al., 2019; Paquette et al., 2018). Mistrust of the healthcare system among socially complex patients has also been specifically shown to discourage participation in self-management programmes provided through said system, likely because such patients expect to feel judged or powerless (Goodridge et al., 2019). Given that seeking care is itself a self-care action, it needs to be encouraged by reducing the access barriers that marginalized PWUD face, such as stigma (Lago et al., 2020). While some PWUD attempt to counter healthcare stigma through self-advocacy, this is unlikely to be either possible or effective for most marginalized people given their low-power social status. Thus, an essential part of community workers’ (including peers’) roles may be providing advocacy support. Healthcare providers need to consider PWUD’s relational autonomy as a key part of delivering patient-centred care, recognizing the structural factors that limit their capacity to self-manage and using this conceptualization to better support their autonomy and thus self-management (Ells et al., 2011; Ould Brahim, 2019). Specialized training may be required to enhance tailored responses for this population.

All three themes are intricately interconnected and demonstrate how substantial social and economic barriers overpower the limited facilitators or supports participants accessed in their attempts to self-manage health issues. Overall, the environmental constraints that participants outlined demonstrate a great need for structural interventions that address resource inequities to support chronic disease self-management among marginalized PWUD. Moreover, our findings align with four identified types of resources that social networks can provide, namely: “social support, social influence, social engagement and attachment, and access to resources and material goods” (Berkman et al., 2000; Tausig, 2013). As Tausig (2013) emphasizes: “It is precisely these resources that determine the quality of ‘self’-management of chronic illness”.

Our findings also highlight some potential solutions for improving the conditions for self-management among this population. Supporting peer leadership is imperative for self-management initiatives among marginalized PWUD, especially to address pervasive anti-drug stigma as well as other socioeconomic concerns. Further, our findings indicate that group-based self-management supports could benefit from selecting participants who share similar experiences to reduce the likelihood of stigmatizing interactions. In addition, the predominant influence of social interactions, demonstrated across all three themes, points to the importance of measuring these types of outcomes (e.g., social support, social capital, social roles, stigma) to assess the utility of self-management supports, as these have been identified as essential but neglected outcomes among other populations with chronic conditions (McDonald et al., 2016; Packer et al., 2018). Common goals of long-term condition management initiatives tend to centre on biomedical and health behavioural outcomes, thus may not reflect patients’ everyday lived realities and priorities, especially among marginalized groups. Our findings highlight the need for current public health self-management initiatives that focus more on social and economic contexts, supporting the use of a relational autonomy lens and enhanced attention to health inequities.

The primary strength of this research was the meaningful community engagement achieved through our commitment to participatory methods, which helped to break down the power imbalance that marginalized groups experience and facilitated the smooth conduct of study activities. For instance, we did not experience difficulties in recruitment, despite the need for participants to identify as people who engage in highly stigmatized activities such as drug use. Similarly, despite purposely avoiding direct questions about traumatic experiences, many participants chose to share details on such sensitive topics. We attribute this success to the skilled community researchers who were able to quickly establish trust and lend their credibility to the academic interviewer, resulting in a rich dataset that provided new insights into the oft-hidden lifeworld of marginalized PWUD. Further, the community researchers provided essential support to qualitative analysis and interpretation, especially through sharing their specific local and cultural knowledge, which contextualized and contributed to making sense of the data. However, it is also important to recognize the need to dedicate sufficient additional time and maintain flexibility to ensure that community engagement is truly meaningful, especially with respect to community participation in the analysis process, which is often the least common stage of a research study to involve community members (Flicker & Nixon, 2015).

As a consideration for future research, we note that discourse around participants’ life course came up frequently and clearly related to their social and economic resources as well as self-management practices. However, our analysis focused on the present, so fully exploring such connections was out of our scope. We suggest that future self-management research with PWUD or other marginalized groups may benefit from explicitly adopting a life course framework. In addition, given the need for complex systems-level change to support self-management among this population, we suggest future research assess the barriers and facilitators to such change.

## 6. Conclusions

The marginalized PWUD who participated in this study were considerably constrained with respect to chronic disease self-management due to complex challenges in their social and economic environments. Most prominently, they needed to prioritize attending to unstable housing, low income, lack of supportive social networks, and negative healthcare experiences, highlighting the need for structural interventions to support their self-management. Yet, they also sometimes benefited from specific aspects of social interactions, such as close relationships, community connectedness, and helping others. We recommend that chronic illness self-management initiatives embrace a relational autonomy approach to facilitate understanding the experiences of marginalized PWUD and other marginalized groups, so as to ensure addressing the constraints of their social networks, economic circumstances, and power relations. We suggest self-management supports for marginalized PWUD should include many low-barrier community-based options, peer work or mutual support opportunities, and advocacy for needs including systems-level change.
